# Dysregulated gene expression associated with inflammatory and translation pathways in activated monocytes from children with autism spectrum disorder

**DOI:** 10.1038/s41398-021-01766-0

**Published:** 2022-01-26

**Authors:** Heather K. Hughes, Megan E. Rowland, Charity E. Onore, Sally Rogers, Annie Vogel Ciernia, Paul Ashwood

**Affiliations:** 1 Department of Medical Microbiology and Immunology, School of Medicine, University of California, Davis, CA USA; 2 M.I.N.D. Institute, University of California, Davis, CA USA; 3 Department of Biochemistry and Molecular Biology, University of British Columbia, Vancouver, Canada; 4 Djavad Mowafaghian Centre for Brain Health, University of British Columbia, Vancouver, Canada; 5 Department of Psychiatry and Behavioral Sciences, University of California, Davis, CA USA

**Keywords:** Autism spectrum disorders, Diagnostic markers, Scientific community, Physiology, Pathogenesis

## Abstract

Autism spectrum disorder (ASD) is a complex developmental disorder characterized by deficits in social interactions, communication, and stereotypical behaviors. Immune dysfunction is a common co-morbidity seen in ASD, with innate immune activation seen both in the brain and periphery. We previously identified significant differences in peripheral monocyte cytokine responses after stimulation with lipoteichoic acid (LTA) and lipopolysaccharide (LPS), which activate toll-like receptors (TLR)−2 and 4 respectively. However, an unbiased examination of monocyte gene expression in response to these stimulants had not yet been performed. To identify how TLR activation impacts gene expression in ASD monocytes, we isolated peripheral blood monocytes from 26 children diagnosed with autistic disorder (AD) or pervasive developmental disorder—not otherwise specified (PDDNOS) and 22 typically developing (TD) children and cultured them with LTA or LPS for 24 h, then performed RNA sequencing. Activation of both TLR2 and TLR4 induced expression of immune genes, with a subset that were differentially regulated in AD compared to TD samples. In response to LPS, monocytes from AD children showed a unique increase in KEGG pathways and GO terms that include key immune regulator genes. In contrast, monocytes from TD children showed a consistent decrease in expression of genes associated with translation in response to TLR stimulation. This decrease was not observed in AD or PDDNOS monocytes, suggesting a failure to properly downregulate a prolonged immune response in monocytes from children with ASD. As monocytes are involved in early orchestration of the immune response, our findings will help elucidate the mechanisms regulating immune dysfunction in ASD.

## Introduction

Autism spectrum disorder (ASD) is a complex neurodevelopmental disorder (NDD) appearing in early childhood and clinically characterized by impairments in communication, social interactions, and aberrant behaviors such as motor stereotypies. ASD is highly heterogeneous and the degree of deficits and behaviors can vary widely, creating challenges for accurate and early diagnoses [1]. Prevalence of ASD is now estimated to be one in 54 children, with a significant male preponderance [1]. The etiology of ASD is unknown but likely involves a variety of genetic and epigenetic factors influenced by environment interactions [2]. Evidence of immune dysfunction is a recurrent finding that may contribute to ASD severity. Immune links to ASD have been shown pre and postnatally. For instance, familial autoimmunity is highly prevalent, with family history of autoimmune disorders such as Type I diabetes, rheumatoid arthritis, and thyroid disorders significantly increasing the risk of ASD [3, 4]. Immune-mediated disorders are also over-represented in ASD, including higher rates of allergies, asthma, and bowel disorders [5–10], with immune symptoms influencing severity of behaviors [11–13].

Several studies have identified immune dysfunction associated with innate immune responses in ASD. Altered circulating innate immune cells including; cell frequencies, activation status, cellular function, and cytokine production are seen in children with ASD [14–21]. This is often accompanied by increased peripheral levels of cytokines most associated with the innate immune response such as, interleukin (IL)−1β, IL-6, IL-12, IL-8, tumor necrosis factor (TNF)-α, and monocyte chemoattractant protein (MCP)−1 in children with ASD. Immunohistochemistry and protein analysis of post-mortem brain tissue revealed increased inflammatory cytokines and chemokines associated with innate immune activation in brain tissue and cerebral spinal fluid [22–25]. Furthermore, microglia – the resident macrophage-like cells of the brain - exhibited activated phenotypes in ASD prefrontal cortex and increased spatial proximity to neurons [22, 23]. Microglia and perivascular macrophages in ASD post-mortem brain also had increased expression of activation markers [25]. Transcriptome studies have identified dysregulated genes involved in innate immune pathways and signatures of microglial activation in brain tissue [26–29]. Many putative animal models of ASD have shown microglia activation and increased inflammatory cytokines in the brain, suggesting a role of innate cells in neuroinflammation in these models [30–33]. Macrophage activation was also present in ASD-relevant mouse models, with enhanced production of inflammatory cytokines correlating with severity of behaviors including increased grooming and decreased social approach [34, 35].

The innate immune system provides the first line of defense during exposure to pathogens. Monocytes and macrophages are innate sentinel cells that are activated through pattern recognition receptors (PRRs) such as toll-like receptors (TLRs), which broadly recognize conserved microbial motifs called pathogen-associated molecular patterns (PAMPs). TLR engagement leads to activation of transcription factors such as NF-κB and production of proinflammatory cytokines. These early responses then drive downstream immune responses, including antigen presentation and cytokine signaling to activate cells of the adaptive immune system [36]. Innate immune cells thus play a critical role in orchestrating immune responses through production of inflammatory mediators and activation of adaptive immunity, whereby aberrant increased responses in this process can lead to chronic inflammation [37]. We hypothesize that alterations in innate immunity may be contributing to the immune dysfunction frequently seen in ASD.

We previously reported increased inflammatory cytokine production after TLR2/4 stimulation of monocytes from children with ASD [16]; these data support evidence of altered innate immune responses in ASD and were linked to symptom severity. These findings led us to investigate the impact of TLR2/4 activation, with lipoteichoic acid (LTA) and lipopolysaccharide (LPS) respectively, on gene expression in monocytes from children with autistic disorder (AD) and pervasive development disorder—not otherwise specified (PDDNOS) compared to typically developing children (TD). We found that AD monocytes were enriched for a pathway associated with inflammatory activation after TLR stimulation. Furthermore, TD monocytes had decreased expression of genes associated with translation regulation after both treatments, however, this decrease was not seen in either AD or PDDNOS monocytes suggesting a failure to regulate translation after innate activation in monocytes from children on the autism spectrum. Results from this study may provide insight into mechanisms contributing to innate immune dysfunction in ASD.

## Materials and methods

### Study participants

48 children ages 4–9 were enrolled as part of the Autism Phenome Project (APP) conducted at the UC Davis M.I.N.D. Institute. Recruitment, behavioral assessments, and study protocols have been previously described in detail [38]. All participants were clinically evaluated prior to 2012 and diagnoses were confirmed by the Autism Diagnostic Interview-Revised (ADI-R) [39] and the Autism Diagnostic Observation Schedule (ADOS) [40] based on the Diagnostic and Statistical Manual of Mental Disorders, Fourth Edition Text Revision (DSM-IVTR) [41]. Participants were grouped after clinical evaluation into (1) typical developing (TD) children [*N* = 22, 18 M/4 F], (2) children diagnosed with AD [*N* = 17, 13 M/4 F]; or (3) children diagnosed with PDDNOS [*N* = 9, 7 M/2 F]. Demographic and clinical characteristics of participants are provided in Table 1. Participants were recruited consecutively and laboratory personnel were blinded to diagnoses. TD children were assessed for achieving development milestones and screened for ASD-like behaviors using the Social Communication Questionnaire (SCQ) and the Mullen’s Scale of Early Learning (MSEL) [42]. Criteria for placement in TD group included scores of <15 on the SCQ and scores within 2 standard deviations of the mean on all subscales of the MSEL. Exclusion criteria for TD children included any known neurological or behavioral problems, developmental delays, speech language impairments, or sibling(s) with ASD. All subjects were screened and excluded for neurological conditions (including seizure disorders, Fragile X or Rett syndrome), psychiatric disorders, or immune-mediated medical conditions (such as celiac disease or other autoimmune disorder). Participants were also excluded if taking prescription medication, had reported illness or fever within 48 h of blood draw, or had suspected or known vision or hearing problems. Participants were assessed for gastrointestinal symptoms through parent survey at time of recruitment by completing the Childhood Autism Risk from Genetics and Environment (CHARGE) study GI symptom survey, which rates GI symptoms on a Likert scale with (0) = never and (4) = always. This survey is based on the Rome III Diagnostic Questionnaire for the Pediatric Functional GI Disorders [43]. Written and informed consent was obtained from parent prior to participation, and approval for this study was approved by the institutional review boards at the University of California, Davis.

**Table 1:** Clinical characteristics and demographics of study participants.

	TD (*n* = 22)		AD (*n* = 17)		PDDNOS (*n* = 9)	
M/F	18/4		13/4		7/2	
	Mean ± SE	Range	Mean ± SE	Range	Mean ± SE	Range
Age	5.7 ± 0.3	4.5-9.2	5.7 ± 0.2	4.7-7.4	6.6 ± 0.4	4.7-7.9
MSEL DQ	105.0 ± 2.5	80.3-129.6	42.5 ± 4.8	19.7-88.7	67.7 ± 8.5	34.5-117
MSEL VDQ	103.6 ± 2.8	79.2-129.7	70.1 ± 4.0	41.3-111.3	72.3 ± 6.5	48.7-107.1
MSEL NVDQ	103.8 ± 2.2	82.3-126.9	56.3 ± 4.1	36.2-100.0	70.0 ± 7.2	43.1-107.4
ADOS RBB	NA	NA	4.6 ± 0.4	2-8	4.0 ± 0.6	1-6
ADOS SA	NA	NA	15.1 ± 3.2	9-20	11.0 ± 3.6	5-16
ADOS Severity	NA	NA	8.4 ± 1.7	5-10	6.4 ± 1.7	4-10

	TD (*n* = 22)		AD (*n* = 17)		PDDNOS (*n* = 9)	
GI scores (*n*=)	0/1	2+	0/1	2+	0/1	2+
Constipation	22	0	10	7	7	2
Diarrhea	21	1	14	3	8	1
Both (D+C)	22	0	13	4	8	1

*AD* Autistic Disorder, *ADOS* Autism Diagnostic Observation Schedule, *DQ* Developmental Quotient, *MSEL* Mullen Scales of Early Learning, *NDQ* Nonverbal Developmental Quotient, *PDDNOS* Pervasive Developmental Disorder Not Otherwise Specified, *RBB* Restricted and Repetitive Behavior, *SA* Social Affect, *TD* Typically Developing, *VDQ* Verbal Developmental Quotient.

### CD14^+^ cell isolation

To isolate peripheral monocytes, blood was collected into acid-citrate-dextrose Vacutainer tubes (BD Biosciences; San Jose, CA). Tubes were centrifuged for 10 min at 1100x*g* to separate plasma, which was removed and stored at −80 °C. The remaining blood was diluted 1:1 with Hanks Balanced Salt Solution without Ca^2+^ or Mg^2+^ (HBSS; Gibco, Gaithersburg, MD), then layered over a Ficoll-Paque gradient (Pharmacia Biotech, Piscataway, NJ) and centrifuged at room temperature for 30 m at 630 × *g*. Peripheral blood mononuclear cells (PBMC) were harvested by pipetting the interface layer and transferring to a new container where cells were then washed twice with HBSS. CD14^+^ cells were isolated using anti-CD14 magnetic beads according to the manufactures protocol (Miltenyi). Live versus dead cell numbers were determined using trypan blue exclusion.

### Cellular stimulations

CD14^+^ monocytes were seeded in RPMI-1640 (Gibco) with 10% heat-inactivated fetal bovine serum (Omega Scientific; Tarzana, CA), 100 IU/ml penicillin, and 100 IU/ml streptomycin (Sigma, St Louis, MO) at 1.0 × 10^5^ cells/well in 96-well plates. Non-treated (NT) cells served as baseline, treated cells were stimulated with either 1 µg/mL LPS (Escherichia coli serotype 0111:B4, Sigma, St. Louis, MO), or 10 µg/mL LTA (Staphylococcus aureus, Sigma). Cells were cultured at 37 °C with 5% CO_2_. After 24 h, cells were centrifuged at 870x*g* for 10 m and processed for RNA extraction.

### RNA extraction and library preparation

After removal of supernatants, cells were collected in Qiagen lysis buffer using the Qiagen RNeasy Mini Kit (Germantown, MD) following the manufacturer’s instructions. Cells were lysed and homogenized in lysis reagent using QIAshredder homogenizers. 70% ethanol was added to the lysate prior to passing through a spin column at 8000 × *g* to remove genomic DNA. Spin columns containing RNA were washed with ethanol plus RPE buffer three times and centrifuged at 8000 × *g*, then RNA was eluted from the column in 30 uL RNAse-free water. RNA was treated with DNase and concentrated (Zymo Research, Cat No R1014). RNA quality was assessed by bioanalyzer and all samples had RNA Integrity Numbers > 6. QuantSeq 3′mRNA sequencing FWD (RNAseq) libraries (Lexogen, Cat 015.96) were prepared as stated in the Lexogen manual using 30 ng RNA per sample and 18 cycles of PCR amplification. 48 unique, dual-index barcodes were used then samples were pooled, exonuclease VII treated, and sequenced across three HiSeq4000 lanes to generate Single-End 100 base pair reads.

### RNA-Seq analysis

Raw sequencing reads were assessed for quality control using FastQC and multiqc, then trimmed to remove adapter contamination, polyA read through and low-quality reads using BBDuk. All samples were then re-assessed for quality using FastQC and multiqc. Following trimming and quality assessment, reads were concatenated for each sample down to a single fastq file. Samples were aligned to human genome (GRCh38.p12) using STAR with -outFilterMultimapNmax 20 --alignSJoverhangMin 8 --alignSJDBoverhangMin 1 --outFilterMismatchNmax 999 --outFilterMismatchNoverLmax 0.1 --alignIntronMin 20 --alignIntronMax 1000000 --alignMatesGapMax 1000000 settings.

Alignment bam files were then compared using multiqc, indexed using samtools and then reads per ensembl gene from GRCh38.94 were counted using FeatureCounts at the gene id level with strand information. Counts were then assessed for overall quality using multiqc and read into R for statistical analysis using EdgeR and LimmaVoom. To remove low-expressing genes, genes with less than 1 count per million (CPM) reads in at least ¼ of the total libraries (109) were removed from the analysis (Fig. S1). The remaining 12438 genes were then normalized using Trimmed Mean of M-values (TMM) to correct for library composition using calcNormFactors, method = TMM (Fig. S2). The resulting CPM values were then fed into Voom using a design matrix with factors for Diagnosis x Treatment and sex as a covariate. Within subjects correlation for treatment was removed using the duplicateCorrelation function from Voom followed by inclusion of the correlation matrix in lmFit and subject identification as a blocking factor. Individual contrast comparisons were then called using contrasts.fit followed by eBayes. To evaluate the contribution of each factor to the overall gene expression profile, Multi-Dimensional Scaling plots were constructed for diagnosis, treatment, and sex (Fig. S3).

Differentially expressed genes (DEGs) were identified for each comparison of interest using the treat function with a log2foldchange = log2(2) and the decideTests function with a Benjamini Hochberg (BH) correction and method = separate. This produces a list of DEGs for each comparison that both passes BH correction to an adjusted *p*-value of <0.05 and log2 fold change greater than + or – log2(2). Gene list overlaps were visualized using VennDiagram and nVennR and assessed statistically using a one-tailed Fisher’s Exact Test for enrichment with False Discovery Rate (FDR) correction to *p* = 0.05. Gene Ontology (GO) and Pathway enrichment testing were performed using ClusterProfiler against a background universe of all genes expressed (passed filtering) in the experiment with FDR correction to *p* = 0.05. GO term enrichments were simplified using the simplify function in ClusterProfiler to a cutoff of 0.7 using the min function. Heatmaps were made using the pheatmap package. Gene enrichment testing between DEGs and published gene lists were performed using a one-tailed Fisher’s exact test with multiple testing correction (FDR). Spearman’s Rank correlations were performed between log2CPM values and behavioral and GI scores with a Benjamini Hochberg (BH) correction.

## Results

**Fig. 1:**
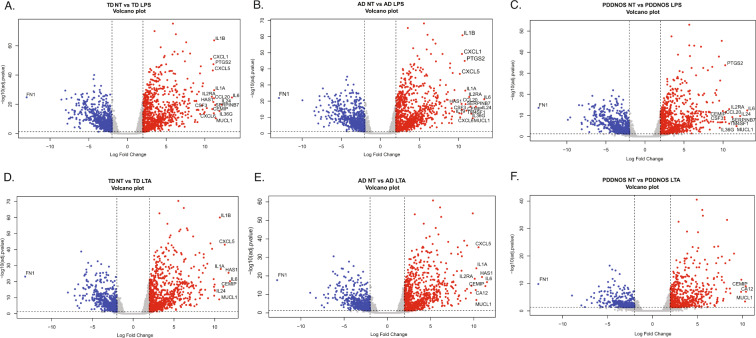
Differentially expressed genes in response to LPS or LTA stimulations. **A** Volcano plot of DEGs for TD NT compared to TD LPS. **B** Volcano plot of DEGs for AD NT compared to AD LPS. **C** Volcano plot of DEGs for PDDNOS NT compared to PDDNOS LPS. **D** Volcano plot of DEGs for TD NT compared to TD LTA. **E** Volcano plot of DEGs for AD NT compared to AD LTA. **F** Volcano plot of DEGs for PDDNOS NT compared to PDDNOS LTA. For A-F genes in red are significantly upregulated with a Log2 Fold Change greater than 2 and genes in blue are significantly downregulated with a Log2 Fold Change less than 2. Genes with a Log2 Fold Change greater or less than 10 are labeled. AD: autistic disorder, TD: typical developing controls, PDD/NOS: pervasive development disorder not otherwise specified, NT: no treatment, LPS: lipopolysaccharide, LTA: lipoteichoic acid.

There were no significant DEGs at baseline (NT) between TD, AD or PDDNOS, indicating that at rest monocytes are similar across diagnoses (Table S1). We then examined the response to LPS or LTA stimulation within each diagnosis. In response to LPS or LTA stimulation (compared to NT samples of the same diagnosis), there were a large number of both increased and decreased DEGs (Fig. 1, Table S1 and S2). These stimulant-responsive genes showed similar overall patterns for TD, AD and PDDNOS monocytes (Fisher’s Exact Test, Table S3). As expected, the major KEGG pathway and GO term enrichment were involved in monocyte activation and were similar across the different diagnoses for both treatments (Table S4). Some of the most common KEGG pathways included cytokine-cytokine receptor interaction, TNF, JAK-STAT, and chemokine signaling pathways (Fig. S4). For Biological Process, terms involving immune activation, inflammatory response, cell adhesion, and chemotaxis were commonly enriched (Fig. S5). In addition, the top shared Molecular Function terms included signaling receptor and molecular transducer activity (Fig. S6). For the analysis of Cellular Component, the integral component of the plasma membrane, cell surface, and extracellular matrix were enriched across DEGs for all three groups and both stimulation types. (Fig. S7).

**Fig. 2:**
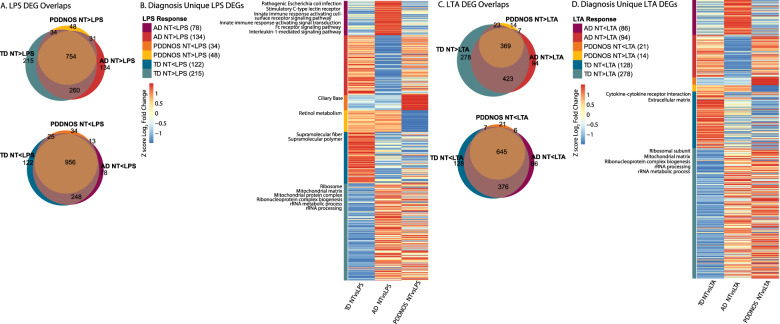
Differentially expressed genes unique to diagnosis conditions. **A** Euler plots showing the overlap among DEGs identified as upregulated or downregulated in response to LPS for each diagnosis. **B** Heatmap of Z-score Log2 Fold Change values for unique LPS DEGs that were significantly regulated in only AD, PDDNOS, or TD (non-overlapping regions of the Euler plots in A) with significantly enriched GO terms and KEGG pathways aligned to heatmap. See Table S6 for full lists. **C** Euler plots showing the overlap among DEGs identified as upregulated or downregulated in response to LTA for each diagnosis. **D** Heatmap of Z-score Log2 Fold Change values for unique LTA DEGs that were significantly regulated in only AD, PDDNOS or TD (non-overlapping regions of the Euler plots in **C**), with significantly enriched GO terms and KEGG pathways aligned to heatmap. See Table S7 for full lists. AD: autistic disorder, TD: typical developing controls, PDD/NOS: pervasive development disorder not otherwise specified, NT: no treatment, LPS: lipopolysaccharide, LTA: lipoteichoic acid.

Although the major responses in ASD and TD were similar in both treatments, i.e., cellular activation, there were subsets of genes that uniquely responded either only in TD, AD, or PDDNOS monocytes (Fig. 2, Table S5). In response to LPS treatment, 78 genes were uniquely increased in AD monocytes, 34 genes were uniquely increased in PDDNOS monocytes, and 122 in TD monocytes (Fig. 2A). Moreover, 134 genes were uniquely decreased in monocytes from children with AD, 48 genes were uniquely decreased in PDDNOS monocytes, and 215 genes in TD monocytes (Fig. 2A). KEGG pathway enrichment analysis of genes uniquely increased in ASD monocytes after LPS revealed significant enrichment in the pathogenic *E. Coli* infection pathway (Fig. 2B and Table S6), including several key immune regulator genes such as FAS cell surface death receptor (*FAS*), nuclear factor kappa B (*NFKB1*), and TGF beta Kinase 3 (*TAB3*). In contrast, genes that were uniquely increased in response to LPS in TD monocytes were enriched for supramolecular fiber and supramolecular polymer. When exploring differentially decreased genes, those uniquely decreased in TD monocytes after LPS stimulation were enriched for GO terms involving translation regulation processes such as the ribosome, rRNA metabolism, and processing (Fig. 2B and Table S6). These genes were not significantly decreased in AD or PDDNOS monocytes in response to LPS.

**Fig. 3:**
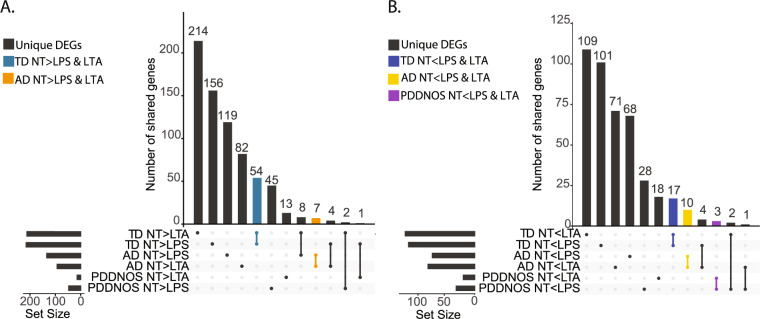
Overlap of unique DEGs for monocyte stimulations in ASD compared to TD. **A** Upset plot of genes that are downregulated in response to LPS or LTA.The majority of genes that are regulated in response to LTA and LPS are not shared within a diagnosis (Gray). Genes with decreased expression in response to both treatments (NT > LPS and LTA) are labeled in blue (TD) and orange (AD). No overlapping genes were present in the PDDNOS group. See Table S8 for statistics and full gene lists. **B** Upset plot of genes that are upregulated in response to LPS or LTA. The majority of genes that are regulated are not shared within a diagnosis (Gray). Genes with increased expression in response to both treatments (NT < LPS & LTA) are labeled in blue (TD), yellow (AD) and purple (PDDNOS). See Table S8 for statistics and full gene lists. AD: autistic disorder, TD: typical developing controls, PDD/NOS: pervasive development disorder not otherwise specified, NT: no treatment, LPS: lipopolysaccharide, LTA: lipoteichoic acid.

After LTA stimulation, 86 genes were uniquely increased in AD monocytes, and 21 genes uniquely increased in PDDNOS monocytes. In contrast, there were 128 genes that were uniquely increased in TD monocytes (Fig. 2C). Of DEGs decreased after LTA stimulation, 94 were uniquely decreased in ASD monocytes, 14 were uniquely decreased in PDDNOS and 278 genes were uniquely decreased in TD (Fig. 2C). Genes that were increased in expression only in ASD were not associated with known KEGG pathways or GO enrichment terms. Genes that were increased in expression only in TD were enriched for cytokine receptor interactions. As with DEGs in TD monocytes after LPS stimulation, the decreased genes in TD monocytes following LTA stimulation were enriched for pathways involved in translation including ribosome, rRNA metabolism and processing (Fig. 2D and Table S7). The majority of genes uniquely decreased in TD did not overlap between the LTA and LPS treatment conditions (Fig. 3). For example, of the genes that uniquely decreased expression in TD, only 54 were shared between LPS and LTA conditions (Fig. 3A). However, these genes contained both the ribosomal and translation-relevant genes identified in the GO term analysis in Fig. 2 (Table S8).

**Fig. 4:**
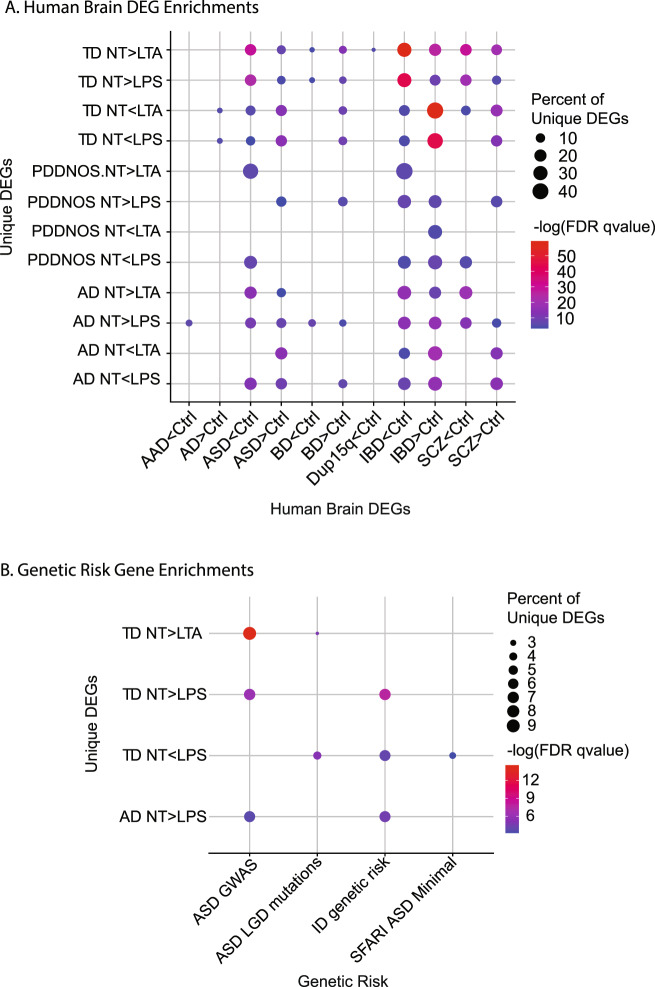
Monocyte stimulated DEGs are enriched for DEGs and genetic risk genes for human brain disorders. **A** Significant enrichments for the overlap between DEGs identified in this study (rows) and published gene lists for DEGS (columns) identified in human brain for neuropsychiatric and neurodevelopmental disorders. Significant enrichments are colored by -log(FDR qvalues) and the percent of unique DEGS shared between the two lists is represented by dot size. BD: bipolar disorder, SCZ: Schizophrenia, AD: Alzheimer’s disease, IBD: inflammatory bowel disease, AAD: alcoholism, Dup15q: duplication 15q syndrome. Gene lists, citations and statistics are in Table S9. **B** Significant enrichments for the overlap between DEGs identified in this study (rows) and published risk genes for ASD and ID (columns). Significant enrichments are colored by -log(FDR qvalues) and the percent of unique DEGs shared between the two lists is listed in each block. Gene lists, citations and statistics are in Table S9.

DEGs from this study were analyzed for shared enrichment with published lists for DEGs in post-mortem human brain from neuropsychiatric and neurodevelopmental disorders (Fig. 4A and Table S9). The profile of DEGs responsive to stimulation in monocytes from this study were significantly enriched in DEGs identified across different human brain disorders or inflammatory diseases, including schizophrenia, Alzheimer’s disease, alcoholism, depression, inflammatory bowel disease (IBD), bipolar disorder, and ASD [44–52]. Some of these overlapping genes had GO enrichment involving protein translation and protein localization (Table S10). DEGs identified in this study were also enriched within identified genetic risk genes for ASD and intellectual disability (ID) (Fig. 4B and Table S9). Together these data suggest that a subset of genes that confer genetic risk for ASD and ID are also critically regulated in monocytes in response to immune stimulation. Gene expression correlations for each condition to behavioral or GI scores are shown in Table S11. Only one of these correlations was seen in a unique DEG after associated stimulation. Expression of *KLHDC7B-DT* which encodes long non-coding RNA not involved in translation [53] correlated with MSEL DQ scores for all participants on the autism spectrum after LTA exposure.

## Discussion

We analyzed gene expression of peripheral monocytes from children with ASD and TD controls that were stimulated with ligands for TLR2/4. There is a strong inflammatory response after activation of TLRs [54], and as expected, our data demonstrate significant changes in expression of subsets of immune genes after stimulation. Although the majority of DEGs overlapped across all diagnostic groups, we also found uniquely expressed genes depending on diagnoses and treatment. An inflammatory KEGG pathway involved in pathogenic *E. coli* responses was increased in monocytes from children with AD after LPS stimulation, in addition to GO biological processes involving innate immune activation. In contrast monocytes from TD showed decreased gene pathways for translation regulation after both treatments. Monocytes from PDD-NOS children did not have similar increases in genes associated with inflammatory pathways and GO processes. However, like AD they did lack a significant decrease in translation associated genes after stimulation as seen in TD. These data may suggest prolonged activation of AD monocytes after stimulation, and a failure in normal down-regulation of translation across monocytes from both AD and PDDNOS. Inflammatory differences between AD and PDDNOS are interesting and may potentially provide insight into the heterogeneity of ASD now that these disorders are grouped together under this umbrella term. In TD monocytes, LPS and LTA stimulation activated different genes but they ultimately converge to control similar pathways and suggest some common and some unique signaling mechanisms that are engaged only in activated TD but not in monocytes from AD or PDD-NOS, specifically in decreased translation in response to stimulation.

Differentially expressed genes have previously been seen in unstimulated peripheral immune cells from children with ASD compared to TD [55, 56]. However, few studies have looked specifically at monocytes in ASD or how these cells respond to activation. Our findings of increased expression of *FAS*, *NFKB1*, and *TAB3* after TLR4 stimulation highlights increased innate immune responses in ASD monocytes, specifically activation of NF-κB which mediates proinflammatory responses [57–60]. *NFKB1* encodes for a subunit of the transcription factor NF-κB [54, 58] and DNA binding by NF-κB leads to production of many inflammatory mediators, including IL-1β, IL-6, and TNFα [58]. NF-κB is also downstream of the phosphoinositide 3-kinase (PI3K)/AKT pathway, which has been implicated previously in ASD and recently found to be overexpressed in leukocytes isolated from ASD toddlers [61, 62]. Increased DNA binding of NF-κB has been seen previously in PBMC of children with ASD [63]. Furthermore, NF-κB was elevated in post-mortem brain of ASD and localized to microglia, astroglia and neurons [64]. Increased IKK*α* kinase, a subunit of the enzyme complex that assists in activating NF-κB, was also seen in the post-mortem ASD brain. However, there were no differences in protein expression or phosphorylation of NF-κB [65]. Recently, monocytes from children with ASD were found to have increased IL-17RA expression, and signaling through this receptor led to NF-κB phosphorylation and increased inducible nitric oxide synthase (iNOS) production [17].

After activation, monocytes from TD children showed decreased expression of genes involved in protein synthesis and translation, changes not observed in monocytes from children on the autism spectrum, regardless of diagnosis. Controlling protein levels is essential for proper function of cells, and many intrinsic mechanisms exist for regulating translation in response to cellular stressors [66, 67]. By limiting the rate of general translation initiation and increasing translation of specific mRNAs, the cell is able to reprogram gene expression to maintain proteostasis after an integrated stress response [68]. In this study, monocytes from TD individuals decreased expression of protein translational machinery after both treatments. Translation genes decreased after LPS, LTA, or both treatments include eukaryotic initiation factors (*EIF2D* and *EIF3E*). In addition, ribosomal biogenesis was significantly decreased in stimulated TD monocytes, including expression of genes that code for several ribosomal proteins and mitochondrial ribosomal proteins. Composition and abundance of ribosomes are recognized to be involved in the regulation of protein translation [66].

In striking contrast, monocytes from children with AD and PDDNOS did not exhibit significant decreases in expression of genes that encode translational machinery. Genetic mutations leading to dysregulated pathways of protein synthesis are common in monogenic forms of NDD such as Fragile X syndrome, tuberous sclerosis (TSC) type 1 and 2, and Cowden syndrome [69]. For example, in Fragile X the *FMR1* gene is silenced, preventing production of FMRP, an RNA-binding protein involved in repression of translation [70, 71]. Altered expression of translational machinery has also been implicated in animal models of ASD. For example, overexpressing the translation initiation factor eIF4E in mice induced autistic-like behaviors and pathophysiological changes to synapses in the brain [72]. Furthermore, conditional overexpression of eIF4E in microglia alone, but not neurons or astrocytes, led to ASD-like behaviors in male mice suggesting that dysregulated translation in innate immune cells in the brain is sufficient to cause behavioral abnormalities [73]. Endogenous binding proteins (4E-BPs) sequester eIF4E, and lack of these proteins increases and dysregulates translation [69, 74]. 4E-BP2 knock-out mice exhibited social deficits and repetitive behaviors, and inhibition of eIF4E activity was sufficient to reverse social deficits [75]. Taken together, these studies suggest that dysregulated translation and protein synthesis may be a mechanism that contributes to ASD.

We next sought to identify any overlap of genes uniquely regulated in TD, AD, and PDDNOS monocytes during TLR activation with dysregulated genes across published brain lists. DEGs in the ASD brain have significant overlap with genes involved in mitochondrial translational regulation and cellular protein complex disassembly. Overlap analyses also highlighted several ASD risk genes identified in the SFARI dataset. Interestingly, increased genes associated with innate immune cell activation after TLR4 stimulation overlapped with increased immune activation processes in brains from inflammatory bowel disease (IBD). Psychiatric co-morbidities are common in IBD [76] with increasing evidence in animal models that microglia activation and neuroinflammation may accompany these disorders [77, 78]. Psychiatric disorders are also associated with intestinal barrier dysfunction and altered gut microbiota (dysbiosis), which may influence the gut-brain axis and contribute to the psychiatric issues seen in IBD [79]. Gut dysfunction and dysbiosis is also a common co-morbidity in ASD [80] and our findings may suggest a shared mechanism in brain disorders and inflammatory gut diseases.

Despite the significant results obtained, this study had several limitations including a relatively small sample size, a single time point after stimulation, and RNA sequencing approach. We chose the CD14^+^ isolation and the timing of stimulation to align with our previous study [16]. At 24 h, we may be missing early responses, or later regulatory responses may be camouflaging early dysregulated gene expression. Based on our findings, further time-course studies and/or stimulation with varying concentrations warrant investigation. We chose to identify differential expression using QuantSeq which sequences only the 3′ end of mRNA. This approach allows for quantitative differential gene analysis with the advantage of lower sequencing costs. However, this approach does not allow for the analysis of differential splicing, differential transcript usage or the analysis of non-coding RNAs. The levels of measured transcripts could also be influenced by RNA stability and degradation changes; additionally, transcript levels do not always reflect functional protein differences. Future work could explore how differences in expression of the translation machinery genes impact protein expression.

To our knowledge, this is the first investigation of gene expression after stimulation of monocytes from children with ASD. We identified a unique inflammatory signature in LPS-stimulated monocytes from children with AD when compared to TD controls, however, this inflammatory signature was not seen in PDDNOS. Moreover, we found decreased expression of genes for protein synthesis and transcriptional machinery after 24 h of stimulation in TD monocytes. This phenomenon was not seen in the either AD or PDDNOS monocytes, which suggests a failure to down-regulate translation after immune activation in monocytes from children on the autism spectrum, perhaps contributing to a failure in immune resolution/control. DEGs from this study shared some overlap with genes previously found altered in brains of psychiatric and neurodevelopmental disorders, suggesting peripheral gene expression changes mirror at least a subset of changes in the brain. Collectively, these data support a role for innate immune dysfunction in ASD through altered immune activation in a subset of ASD and aberrant translational control in ASD monocytes.

## Supplementary information

Supplementary Figures

Supplemental table legends

Table 1S

Table 2S

Table 3S

Table 4S

Table 5S

Table 6S

Table 7S

Table 8S

Table 9S

Table 10S

Table 11S

## Data Availability

The accession number for the RNA-sequencing raw data reported in this paper is NCBI Gene Expression Omnibus: GSE140702. Code is available at: https://github.com/ciernialab/RNAseq_Ashwood_2021.
