# Supplementary material for: Dysregulated gene expression associated with inflammatory and translation pathways in activated monocytes from children with autism spectrum disorder
Source: Transl Psychiatry. 2022 Jan 26;12:39. doi: 10.1038/s41398-021-01766-0 (PMC8791942; doi:10.1038/s41398-021-01766-0)
Supplement: Supplementary file 1 — Supplementary Figures [file 41398_2021_1766_MOESM1_ESM.pdf]

Supplementary Figures

Figure S1

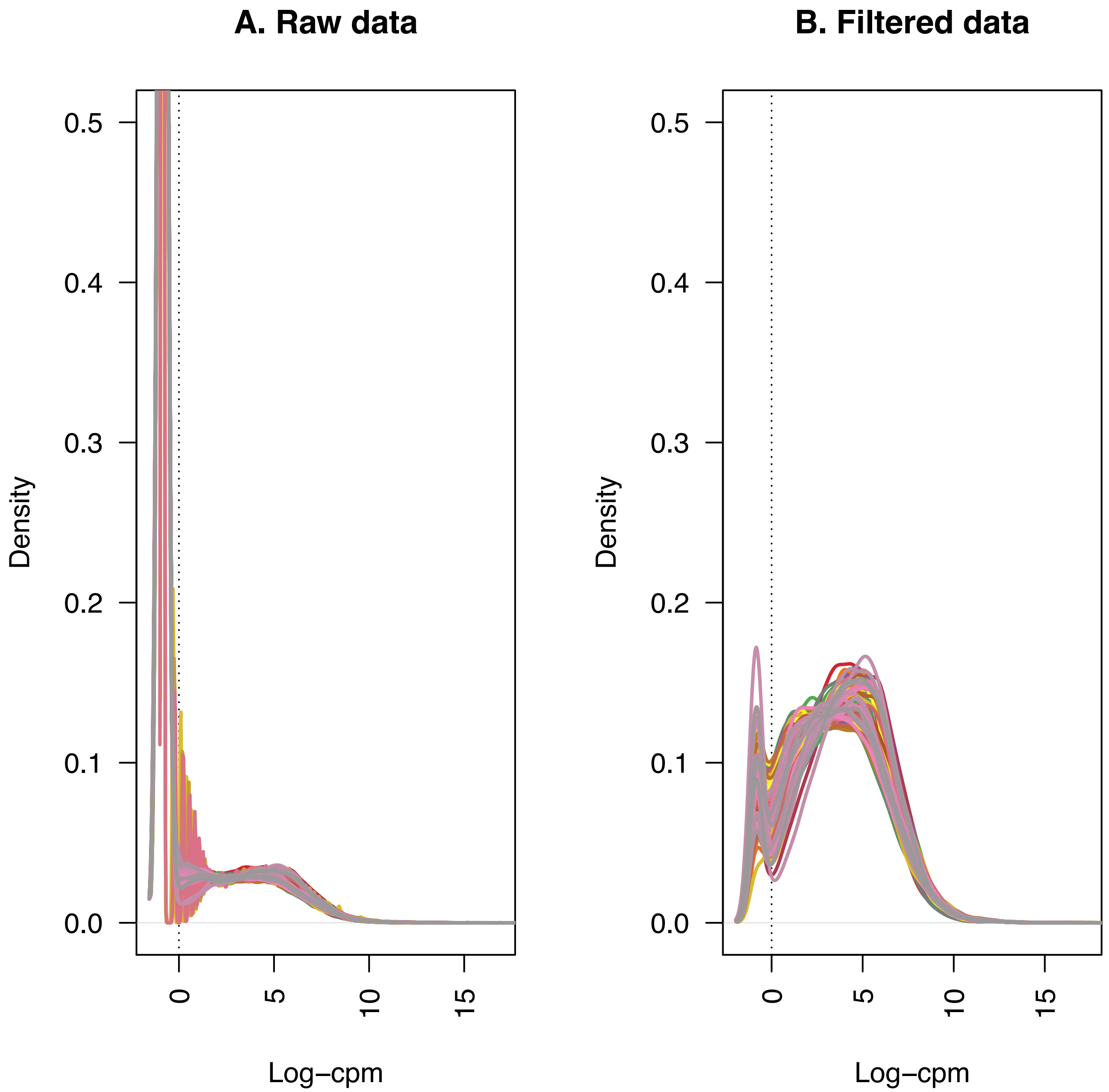

Figure S1 Read Filtering. A. Distribution of log Counts Per Million (CPM) for each sample prior to filtering. Notice that genes with low expression levels are < 0. B. Distribution of log Counts Per Million (CPM) for each sample after filtering to remove lowly expressed genes (CPM < 1 in ¾ of samples).

Figure S2

A.

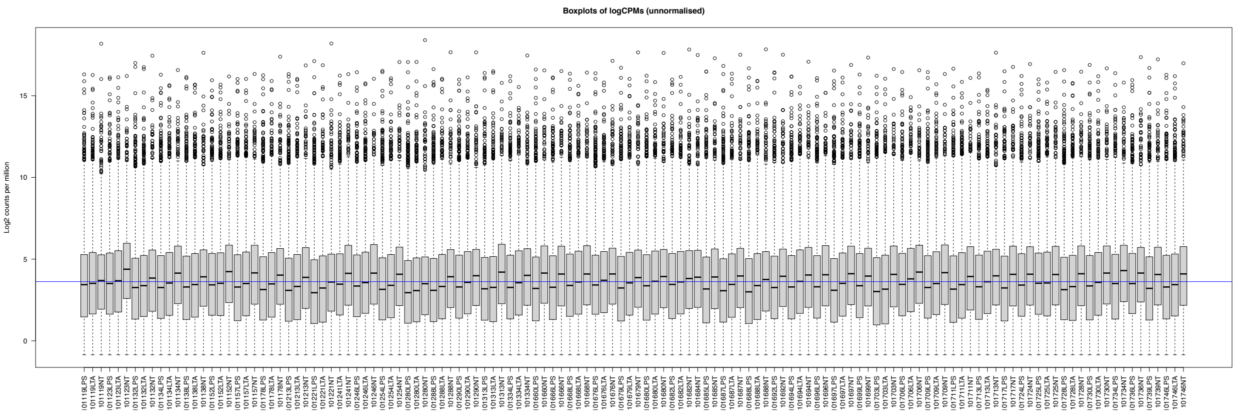
Boxplots of logCPMs (unnormalised)
Log2 counts per million
0
5
10
15

B.

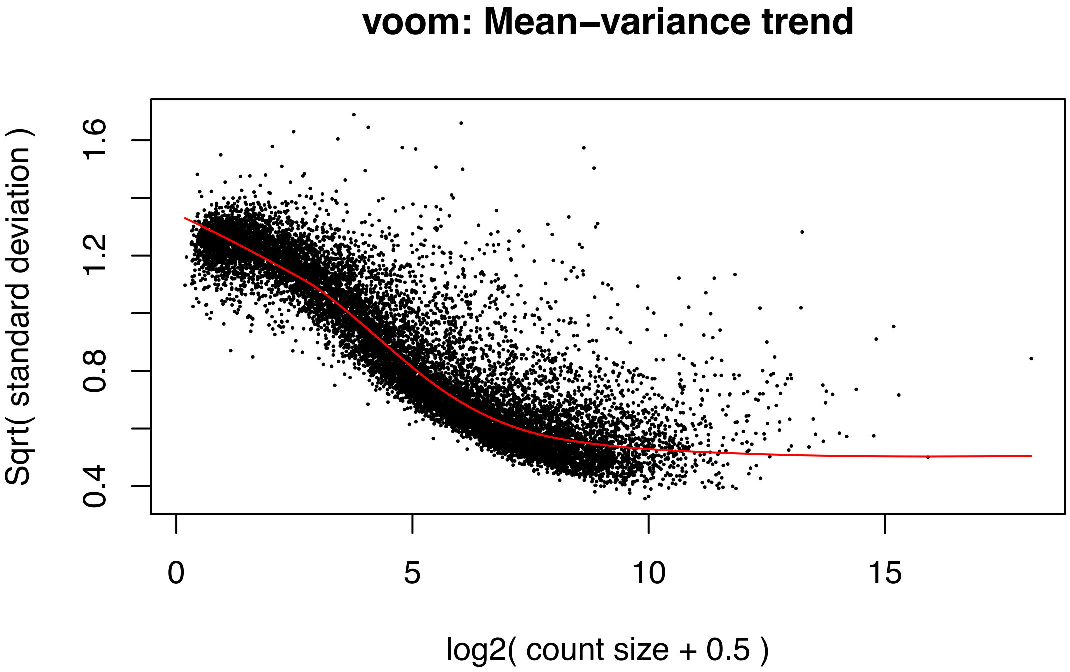
voom: Mean−variance trend
Sqrt(standard deviation)
0.4
0.8
1.2
1.6
0
5
10
15
log2(count size + 0.5)

C.

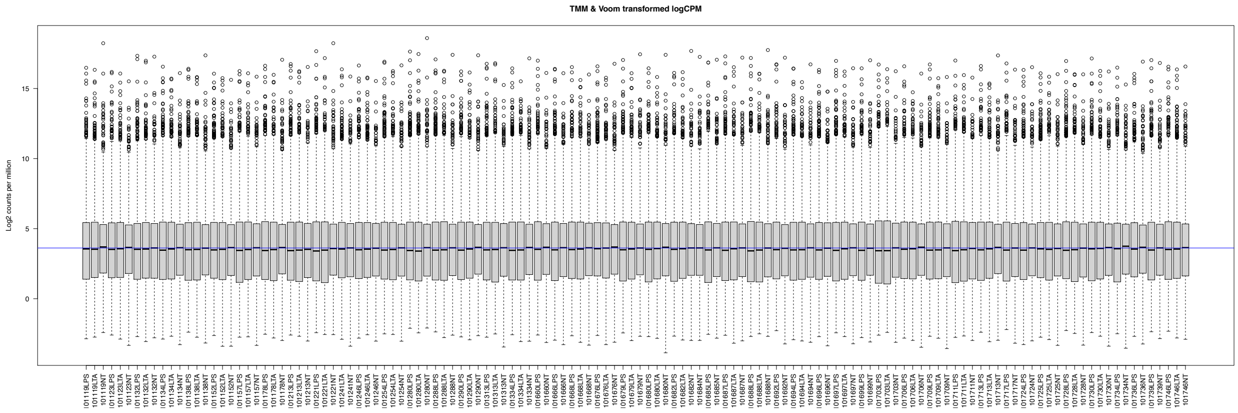

Figure S2 Normalization. A. Boxplots of Log2CPM values for each sample prior to normalization. B. Limma-voom trend line. C. Boxplots of Log2CPM values after TMM and limma-voom normalization.

Figure S3

A.

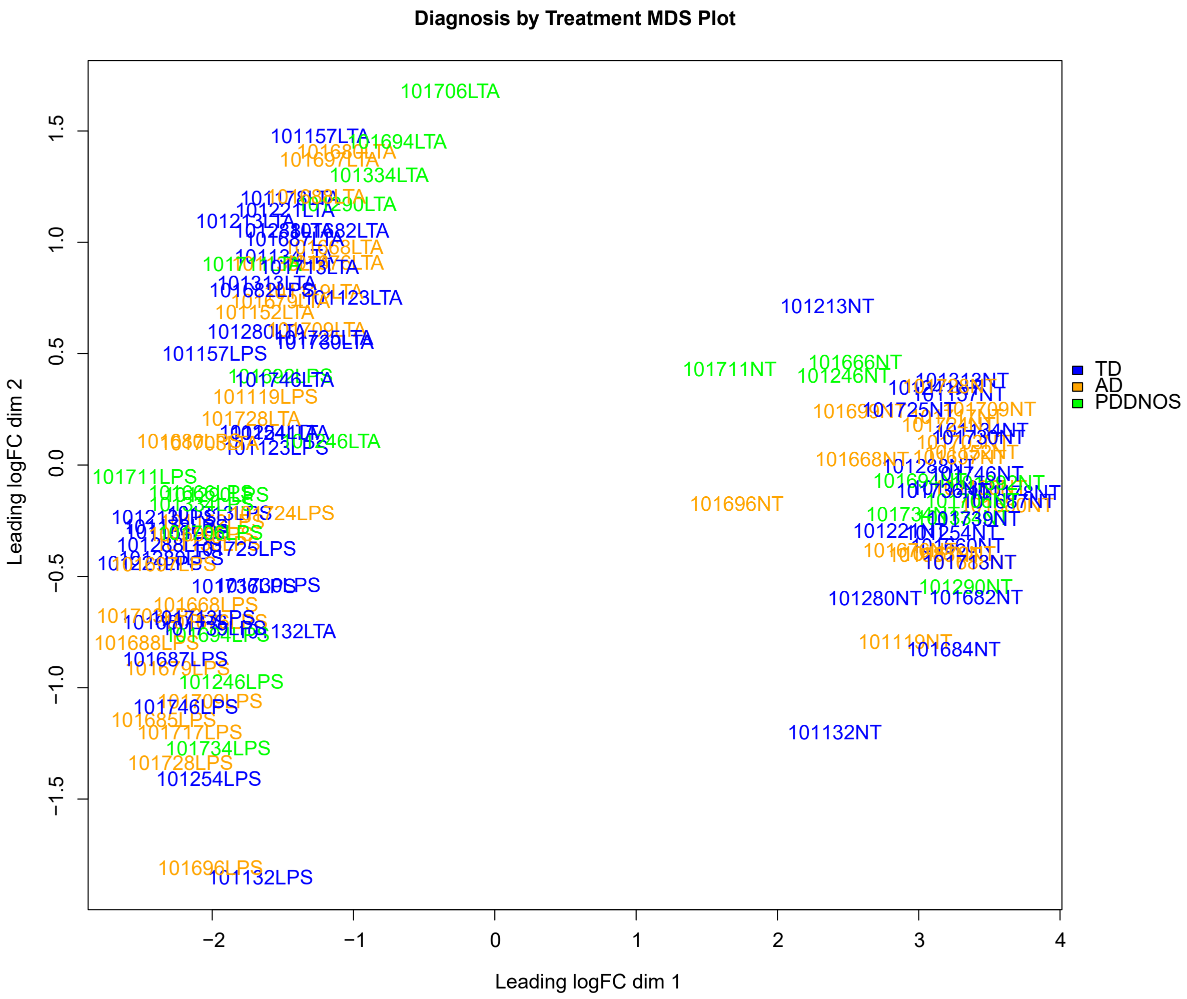
Diagnosis by Treatment MDS Plot
Leading logFC dim 1
Leading logFC dim 2
TD
AD
PDDNOS
101706LTA
101157LTA
101694LTA
101334LTA
101123LTA
101152LTA
101157LPS
101119LPS
101711LPS
101687LPS
101246LPS
101717LPS
101734LPS
101728LPS
101254LPS
101696LPS
101132LPS
101213NT
101711NT
101668NT
101696NT
101280NT
101682NT
101290NT
101684NT
101132NT

B.

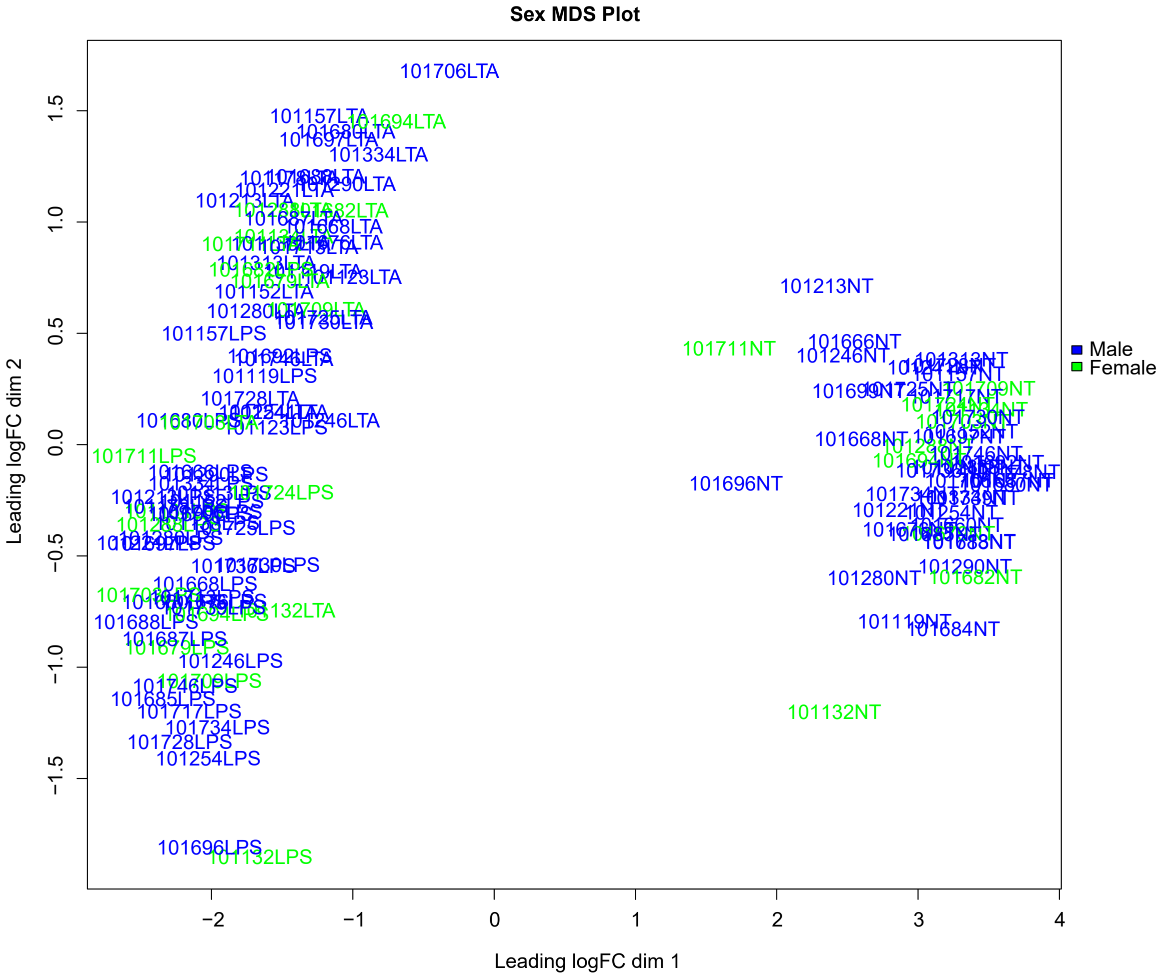
Sex MDS Plot
Leading logFC dim 1
Leading logFC dim 2
Male
Female
−2
−1
0
1
2
3
4
1.5
1.0
0.5
0.0
−0.5
−1.0
−1.5
101706LTA
101157LTA
101694LTA
101334LTA
101152LTA
101157LPS
101119LPS
101728LTA
101711LPS
101213NT
101711NT
101666NT
101246NT
101696NT
101668NT
101280NT
101290NT
101682NT
101119NT
101684NT
101132NT
101668LPS
101688LPS
101687LPS
101246LPS
101685LPS
101717LPS
101734LPS
101728LPS
101254LPS
101696LPS
101132LPS

C.

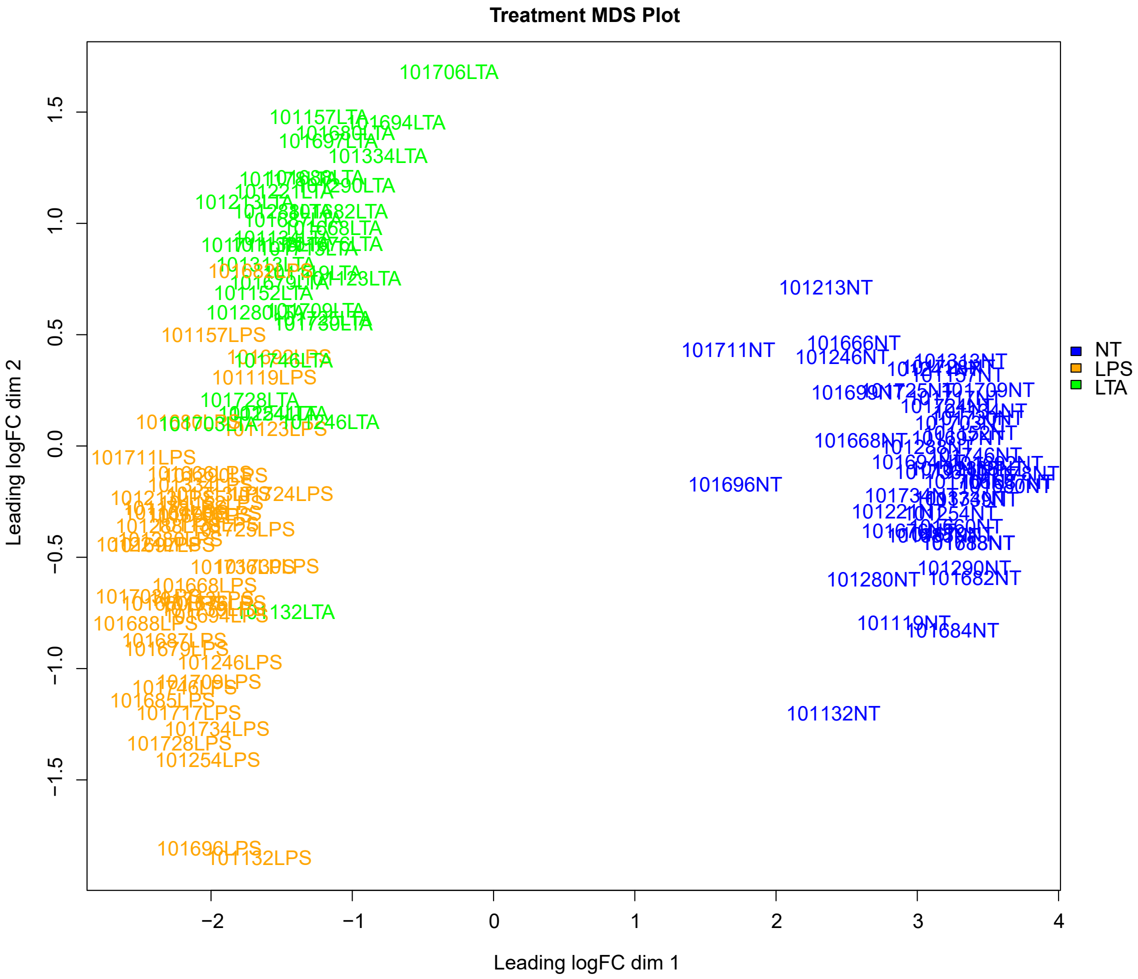

Figure S3 Multi Dimensional Scaling Plots (MDS). A. MDS plot colored by diagnosis. B. MDS plot colored by sex. C. MDS plot colored by treatment.

Figure S4

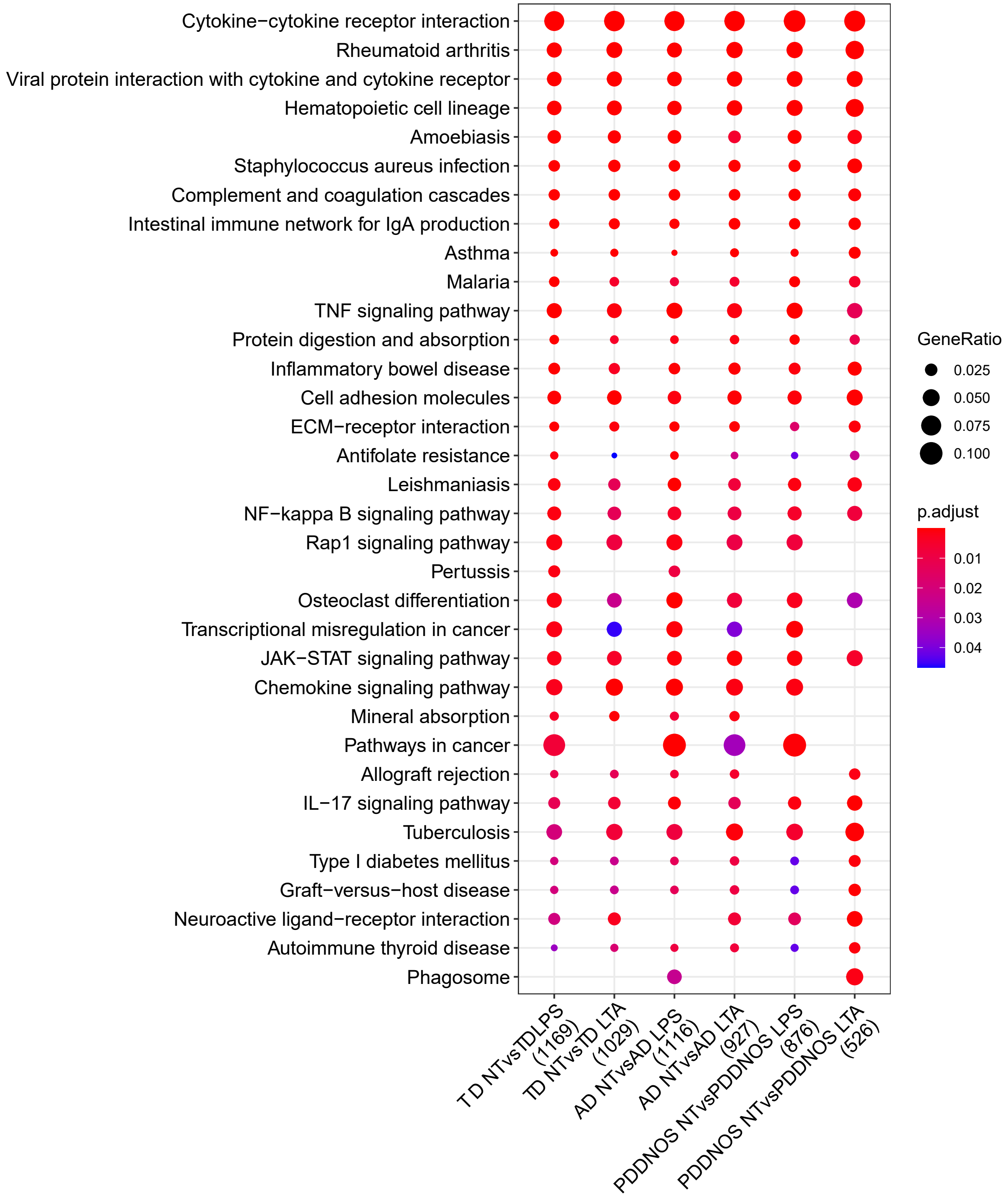

Figure S4 KEGG Enrichments for All DEGs. Top significantly enriched KEGG pathways across lists of DEGs. See Table S4 for full lists and statistics.

Figure S5

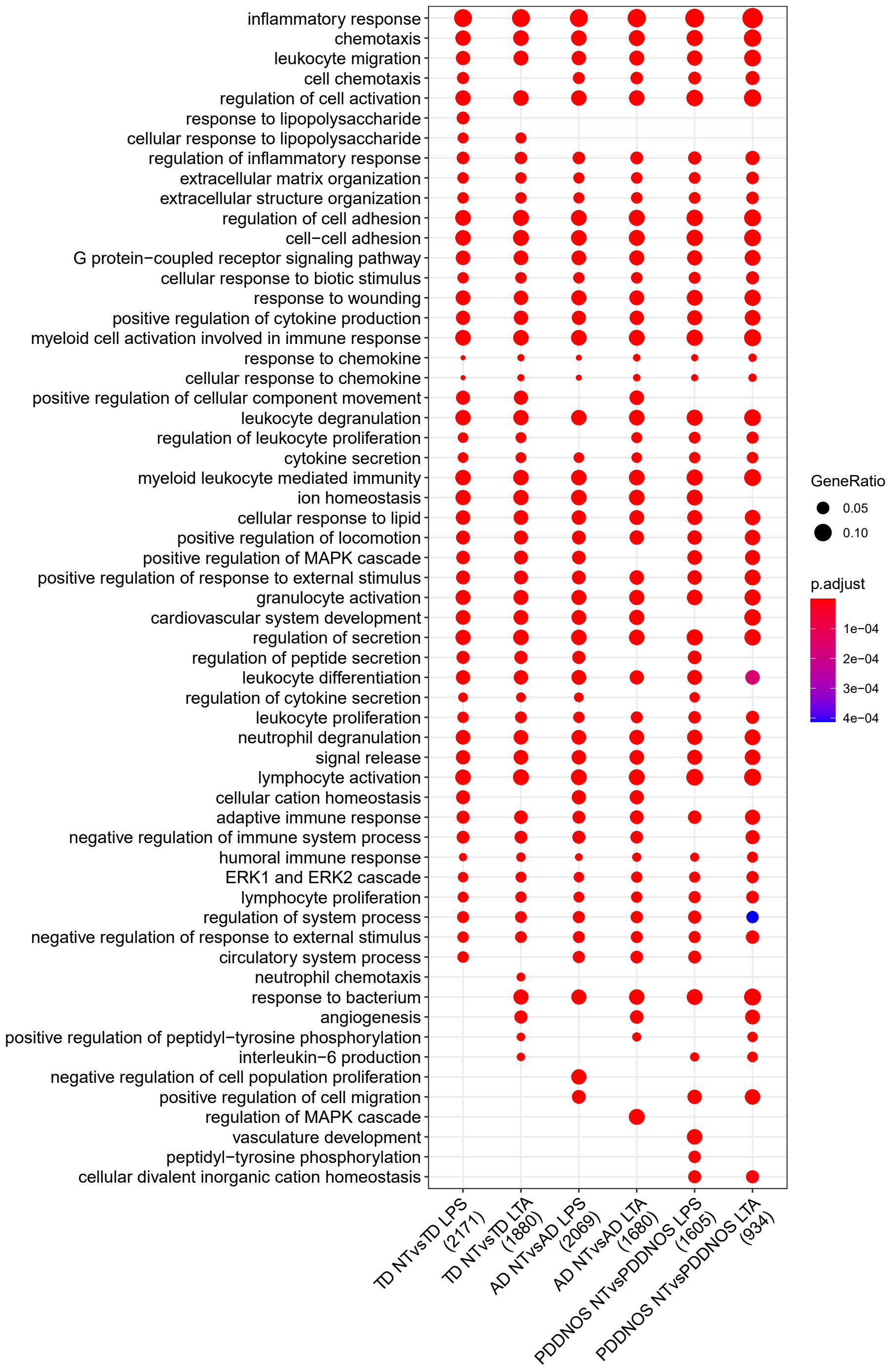

Figure S5 Biological Process GO Term Enrichments for All DEGs. Top significantly enriched Biological Process Terms across lists of DEGs. See Table S4 for full lists and statistics.

Figure S6

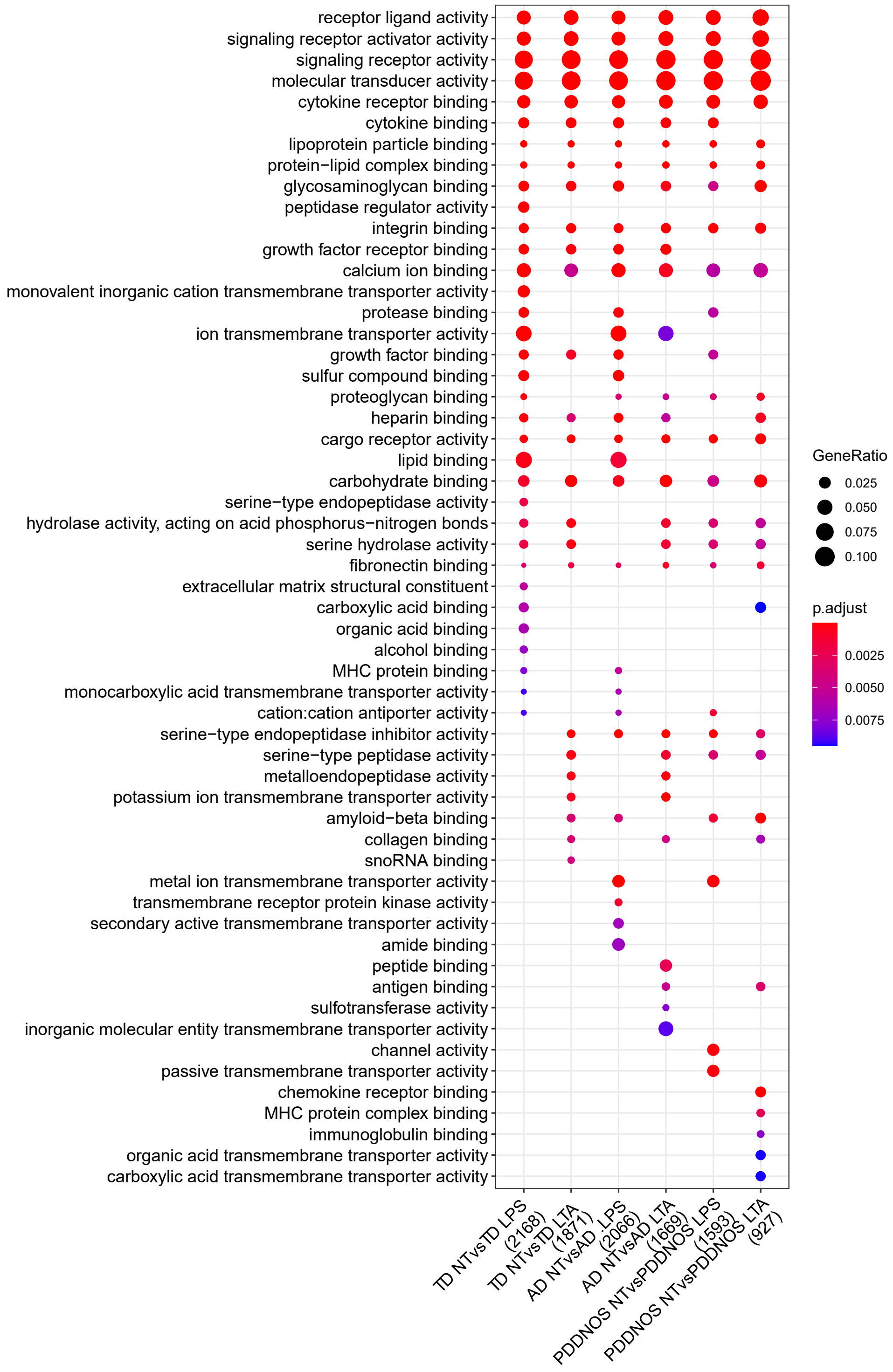

Figure S6 Molecular Function GO Term Enrichments for All DEGs. Top significantly enriched Molecular Function Terms across lists of DEGs. See Table S4 for full lists and statistics.

Figure S7

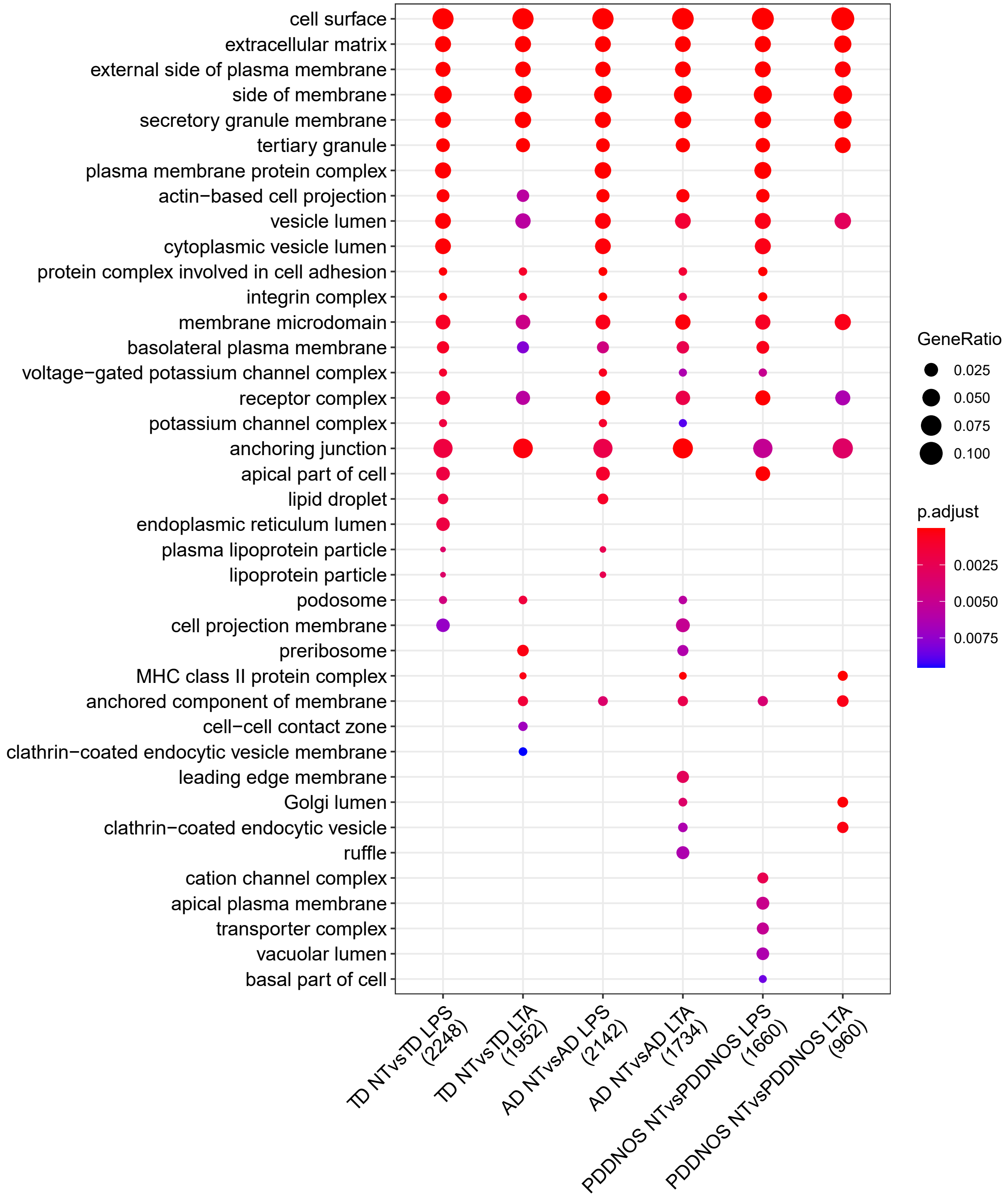

Figure S7 Cellular Component GO Term Enrichments for All DEGs. Top significantly enriched Cellular Component Terms across lists of DEGs. See Table S4 for full lists and statistics.
