# Supplementary material for: Dysregulated gene expression associated with inflammatory and translation pathways in activated monocytes from children with autism spectrum disorder
Source: Transl Psychiatry. 2022 Jan 26;12:39. doi: 10.1038/s41398-021-01766-0 (PMC8791942; doi:10.1038/s41398-021-01766-0)
Supplement: Supplementary file 2 — Supplemental table legends [file 41398_2021_1766_MOESM2_ESM.docx]

***Supplemental Table Legends***

1. Table S1. Summary Count DEGs
   1. Gene counts: number of DEGs for each comparison
2. Table S2. All DEGs for All Conditions
   1. All DEGs: Master output file for all genes in the experiment, significance for each comparison, and membership to LPS or LTA Venn diagrams
3. Table S3. Venn Overlaps for all DEGs
   1. LPS: Fisher’s Exact enrichment testing for LPS DEGs by direction of expression
   2. LTA: Fisher’s Exact enrichment testing for LTA DEGs by direction of expression
   3. All DEGs: Fisher’s Exact enrichment testing for All DEGs, non-directional
4. Table S4. GO Term and Pathway Enrichments for all DEGs
   1. KEGG: Enriched KEGG pathways for each DEG list
   2. MF: Enriched Molecular Function Gene Ontologies for each DEG list
   3. BP: Enriched Biological Process Gene Ontologies for each DEG list
   4. CC: Enriched Cellular Component Gene Ontologies for each DEG list
5. Table S5. DEG Heatmap Genes
   1. LPS DEGs Heatmap: Genes uniquely regulated in one diagnosis in response to LPS treatment. Shown in heatmap in Figure 4A.
   2. LTA DEGs Heatmap: Genes uniquely regulated in one diagnosis in response to LTA treatment. Shown in heatmap in Figure 4B.
   3. Unique DEGs belonging to TD NT>LPS or >LTA
6. Table S6. GO Term and Pathway Enrichments for LPS Heatmap Genes (Figure 4A)
   1. KEGG: Enriched KEGG pathways for genes uniquely regulated in each diagnosis in response to LPS
   2. GO BP: Enriched Biological Process Gene Ontologies for genes uniquely regulated in each diagnosis in response to LPS
   3. GO CC: Enriched Cellular Component Gene Ontologies for genes uniquely regulated in each diagnosis in response to LPS
7. Table S7. GO Term and Pathway Enrichments for LTA Heatmap Genes (Figure 4B)
   1. KEGG: Enriched KEGG pathways for genes uniquely regulated in each diagnosis in response to LTA
   2. GO MF: Enriched Molecular Function Gene Ontologies for genes uniquely regulated in each diagnosis in response to LTA
   3. GO BP: Enriched Biological Process Gene Ontologies for genes uniquely regulated in each diagnosis in response to LTA
   4. GO CC: Enriched Cellular Component Gene Ontologies for genes uniquely regulated in each diagnosis in response to LTA
8. Table S8. Unique DEGs Overlaps
   1. FishersExactTest: Fisher’s Exact Test for overlap between uniquely regulated gene lists for each diagnosis between LPS and LTA treatments
   2. UniqueAD_NT<LPS.NT<LTA: Genes that are uniquely increased in expression only in AD samples (not TD or PDDNOS) in response to LPS and LTA
   3. UniqueAD_NT>LPS.NT>LTA: Genes that are uniquely decreased in expression only in AD samples (not TD or PDDNOS) in response to LPS and LTA
   4. UniquePDDNOS_NT<LPS.NT<LTA: Genes that are uniquely decreased in expression only in PDDNOS samples (not TD or AD) in response to LPS and LTA
   5. UniqueTD _NT<LPS.NT<LTA: Genes that are uniquely increased in

expression only in Typical samples (not AD or PDDNOS) in response to LPS and LTA

- 1. UniqueTD _NT>LPS.NT>LTA: Genes that are uniquely decreased in expression only in Typical samples (not AD or PDDNOS) in response to LPS and LTA

1. Table S9. Overlap Gene List Enrichments

One Tailed Fisher’s exact test for overlap enrichment between DEG lists identified in this study and published gene lists.

- 1. Published Gene Lists: List name, description, and citations for each list included in the enrichment analysis
  2. Significant List Enrichments: Results for significant overlaps (FDR corrected one tailed Fisher’s Exact test).

1. Table S10. GO Term and Pathway Enrichments for Gene List Enrichments
   1. GO Term Enrichment for overlap between DEG lists identified in this study and published gene lists
2. Table S11. Participant Scores Significantly Correlated with Gene Expression
   1. Significant Spearman’s rank correlation between ASD (all AD and PDDNOS) behavior and GI scores and Log2CPM gene expression (FDR corrected). ADOS RBB total: Autism Diagnostic Observation Schedule Restricted and Repetitive Behavior Total, ADOS SA: Autism Diagnostic Observation Schedule Social Affect Total, MSEL DQ: Mullen Scales of Early Learning Developmental Quotient, MSEL NVDQ: Mullen Scales of Early Learning Non-Verbal Developmental Quotient, MSEL VDQ: Mullen Scales of Early Learning Verbal Developmental Quotient.
